# UAV Vision-Based Method for Multi-Point Displacement Measurement of Bridges

**DOI:** 10.3390/s26010240

**Published:** 2025-12-30

**Authors:** Deyong Pan, Wujiao Dai, Lei Xing, Zhiwu Yu, Jun Wu, Yunsheng Zhang

**Affiliations:** 1 National Engineering Research Center of High-Speed Railway Construction Technology, Changsha 410075, China; 2 Department of Surveying Engineering & Geo-Informatics, Central South University, Changsha 410083, China; 3 China Railway Group Limited, Beijing 100039, China

**Keywords:** unmanned aerial vehicle (UAV), vision-based measurement, bridge monitoring, multi-point displacement

## Abstract

**Highlights:**

**What are the main findings?**

- A UAV vision-based multi-point displacement measurement system (integrating a UAV-mounted camera, computing terminal, and targets) is proposed to address accuracy limitations arising from UAV motion interference and camera performance constraints.
- Field tests on Lunzhou Highway Bridge (Guangdong Province) successfully captured full-span vertical multi-point dynamic displacements under traffic loads, with a root mean square error (RMSE) < 0.3 mm—consistent with results from a Scheimpflug camera.

**What are the implications of the main findings?**

- The system’s flexible deployment in complex environments enhances the applicability of high-precision, non-contact technologies for bridge displacement monitoring.
- It provides critical data for understanding bridge deformation behavior, supports reliable safety assessments, and advances UAV vision applications in bridge health monitoring.

**Abstract:**

The challenge of insufficient monitoring accuracy in vision-based multi-point displacement measurement of bridges using Unmanned Aerial Vehicles (UAVs) stems from camera motion interference and the limitations in camera performance. Existing methods for UAV motion correction often fall short of achieving the high precision necessary for effective bridge monitoring, and there is a deficiency of high-performance cameras that can function as adaptive sensors. To address these challenges, this paper proposes a UAV vision-based method for multi-point displacement measurement of bridges and introduces a monitoring system that includes a UAV-mounted camera, a computing terminal, and targets. The proposed technique was applied to monitor the dynamic displacements of the Lunzhou Highway Bridge in Qingyuan City, Guangdong Province, China. The research reveals the deformation behavior of the bridge under vehicle traffic loads. Field test results show that the system can accurately measure vertical multi-point displacements across the entire span of the bridge, with monitoring results closely matching those obtained from a Scheimpflug camera. With a root mean square error (RMSE) of less than 0.3 mm, the proposed method provides essential data necessary for bridge displacement monitoring and safety assessments.

## 1. Introduction

Monitoring bridge structures is a vital aspect of bridge engineering and is significant for ensuring the safe operation of bridges. Structural displacement is a key indicator in monitoring, as it can be used to calculate the static and dynamic characteristics of bridges, such as deflection and modal parameters. This data is crucial for safety assessments and early warning systems for bridge structures [1].

Currently, various technologies are employed for monitoring structural displacements in bridges, including Global Navigation Satellite System (GNSS) [2,3,4], Linear Variable Differential Transformers (LVDTs) [5], strain gauges [6], Image-Assisted Total Stations (IATS) [7,8], and microwave radar interferometers [9]. However, each of these technologies has its limitations in the context of bridge monitoring. For instance, GNSS lacks the accuracy needed to measure the highly dynamic responses of bridge structures; LVDT technology is constrained by measurement distance; and the installation of strain gauges can be time-consuming and labor-intensive. Robotic total stations are widely used because of their high accuracy and non-contact capabilities. Some researchers have combined robotic total stations with image sensors to create IASTs, enabling precise multi-point displacement monitoring [8]. Additionally, microwave radar interferometers, which provide high measurement accuracy and can operate under various weather conditions, have also gained considerable attention in recent years. Nevertheless, both IATS and microwave radar interferometry tend to be relatively expensive, which hinders their widespread adoption for bridge monitoring.

With advancements in computer vision technology, vision-based measurement systems have become increasingly prevalent in structural displacement measurements due to their non-contact operation, highly dynamic response, precision, and ability to perform real-time multi-point measurements [10,11,12,13,14,15]. To attain high-precision displacement measurements of bridge structures using vision sensors, researchers have proposed various methods that yield promising outcomes. For instance, Lee et al. [12] developed a vision-based dynamic displacement measurement system utilizing artificial targets and cameras, which enabled real-time quantification of the parameters. Feng et al. [13] achieved high-precision measurements of railway bridges by employing template-matching techniques alongside artificial targets.

In recent years, visual measurement systems equipped with cameras on unmanned aerial vehicles (UAVs) have emerged as a research hotspot, finding applications in both detection and measurement fields. For instance, Zhang et al. [16] applied UAVs to the detection, inspection, and fault diagnosis of wind turbine blades, focusing on analyzing the advantages, disadvantages, and adaptability of core detection technologies and inspection control algorithms. Bouguettaya et al. [17] utilized UAV imagery for vehicle detection, reviewed relevant deep learning methods, discussed the challenges in detection and corresponding solutions, and provided references for researchers to select suitable methods. Yoon et al. [18,19] obtained structural displacements from processed UAV videos and further derived the actual structural displacements after compensating for camera motion. With UAV platforms, cameras can measure deformable objects at short distances to improve measurement accuracy. However, the overall accuracy of existing UAV vision measurement systems remains low due to two primary issues: first, significant jitter from the UAV platform, which adversely impacts measurement accuracy; and second, the inadequate performance of cameras installed on current UAVs, characterized by low-quality images and low frame rates. Existing efforts to address UAV jitter mainly fall into three categories: (1) High-pass filtering techniques, which separate the UAV’s ego-motion from the target’s dynamic displacement. For example, Hoskere et al. [20] proposed a high-pass filtering method to remove the low-frequency noise caused by the hovering vibration of the UAV, but this method can only be used in the case that the structural natural frequency is much higher than the noise frequency caused by the UAV motion; (2) Motion correction using the UAV’s built-in inertial measurement unit (IMU) to estimate attitude changes for camera motion correction. Riveiro et al. [21] applied an IMU-based system to mitigate UAV-induced measurement errors. Nevertheless, the attitude estimation accuracy of onboard IMUs is generally insufficient for millimeter-level positioning, limiting the broader applicability of this strategy; (3) Camera motion correction based on fixed reference points, which is currently the most widely adopted approach. For instance, Weng et al. [22] employed homography-based perspective transformation using reference targets and validated their method on an elevator tower. However, homography describes a planar projective relationship and becomes inapplicable when reference targets lie on non-coplanar surfaces. To address these limitations, this paper proposes a motion correction framework for the UAV-borne camera by analyzing the 3D motion characteristics of the camera. In addition, the hardware system is reconfigured through the integration of a UAV-mounted camera, a computing terminal, and a UAV. This work aims to provide a novel solution for high-precision monitoring of bridge displacement.

The remainder of this paper is organized as follows. Section 2 details the materials and methods, including the UAV vision-based measurement system and the camera motion correction method. Section 3 presents the Experiment, covering the method validation and the monitoring of Lunzhou Bridge. Section 4 provides a comprehensive discussion of the findings. Finally, Section 5 concludes the paper.

## 2. Materials and Methods

### 2.1. UAV Vision-Based Measurement System

**Figure 1:**
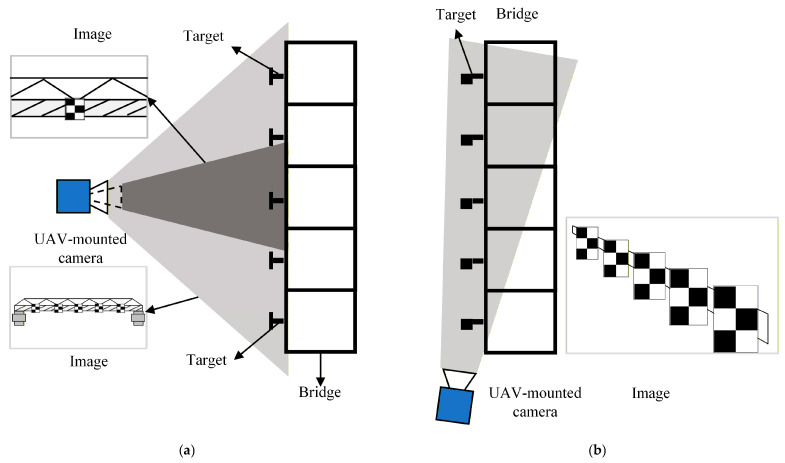
UAV vision-based bridge monitoring configurations: (**a**) lateral view shooting and (**b**) longitudinal view shooting.

There are two primary configurations for applying UAV vision-based measurement technology to multi-point displacement monitoring of bridges, as illustrated in Figure 1. Figure 1a demonstrates the transverse shooting configuration, where the vision sensor is positioned laterally to the bridge, enabling observation of all targets from the side. However, this configuration requires reducing the camera magnification to simultaneously capture multiple targets, consequently diminishing the resolution of targets in the imagery and compromising measurement accuracy. In contrast, Figure 1b shows the longitudinal shooting configuration, where the vision sensor is deployed at either end of the bridge. With proper angle adjustment, all targets remain observable in this arrangement, which proves more conducive to achieving multi-point displacement measurements across the bridge structure.

**Figure 2:**
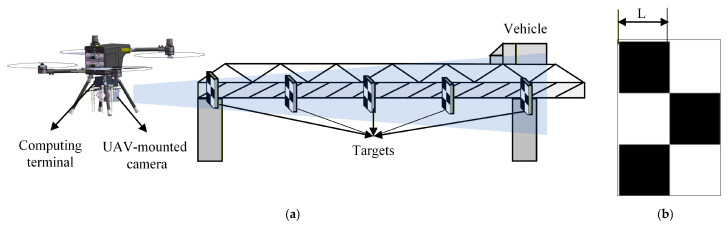

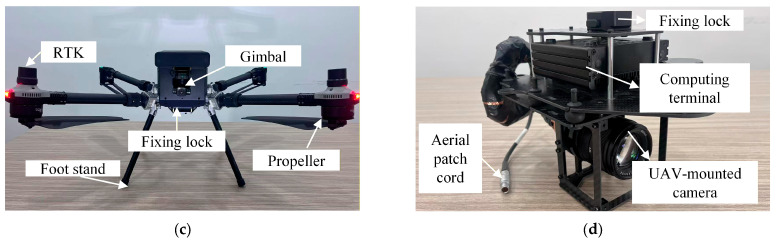
UAV vision-based measurement system. (**a**) Monitoring layout. (**b**) Target. (**c**) UAV. (**d**) Computing terminal and camera.

It should be noted that in the configuration shown in Figure 1b, limitations in the camera’s depth of field may result in blurred target imaging. Therefore, to ensure effective measurements, the UAV generally needs to maintain a considerable distance from the targets to achieve clear imaging. Based on the configuration illustrated in Figure 1b, this paper presents a low-cost, high-precision monitoring system based on UAV vision to measure multi-point displacement of bridges. The system comprises a UAV-mounted camera, a displacement calculation terminal, and targets, as illustrated in Figure 2.

Figure 2a illustrates the overall configuration of the monitoring system (including the UAV, monitoring frame, vehicle, and targets); Figure 2b shows the target (a checkerboard pattern with side length L); Figure 2c displays the UAV, with key components (RTK, gimbal, fixing lock, and foot stand) labeled; and Figure 2d shows the integrated computing terminal and UAV-mounted camera assembly, which is secured by fastening the fixing locks. It is important to note that the system employs a high-performance industrial camera as the image acquisition unit, coupled with a high-capacity computing terminal to achieve high-frequency and high-precision displacement data acquisition. Furthermore, both power supply and communication functions are uniformly provided by the UAV’s built-in battery and router, thereby ensuring operational stability throughout the measurement process. The detailed specifications of this UAV vision-based measurement system are provided in Table 1.

Similar to monocular vision displacement measurement methods, the UAV vision-based method for displacement measurement mainly consists of three steps. Initially, image coordinates are identified. To enhance the stability of the cross-shaped marker positioning method, this paper employs a highly robust detection and positioning technique proposed by Xing [23]. Thereafter, the pixel displacement of the monitored target is calculated by determining the difference in the target’s center across consecutive image sequences. Finally, the pixel displacement is converted into actual displacement by solving for the scale factor. Assuming that both the imaging plane of the camera and the monitored target plane are perpendicular to the horizontal plane, the calculation formula for the vertical scale factor is as follows:

(1) $$SF=\frac{D_{know}}{d_{pixel}}=\frac{S}{f}$$

where SF represent the scale factors, specifically the vertical scale factors $SF_{y}$ and horizontal scale factors $SF_{x}$, respectively. $D_{know}$ denotes the known actual distance, and $d_{pixel}$ is the pixel distance corresponding to this known actual distance. The actual distance between the focal plane and the measured target is represented by S, while f is the focal length of the lens. The mathematical expression for determining the actual displacement is as follows:

(2) $$Disp=SF\times \Delta_{d}$$

where Disp is the actual displacement, and $\Delta_{d}$ is the pixel displacement.

### 2.2. Camera Motion Correction Method

**Figure 3:**
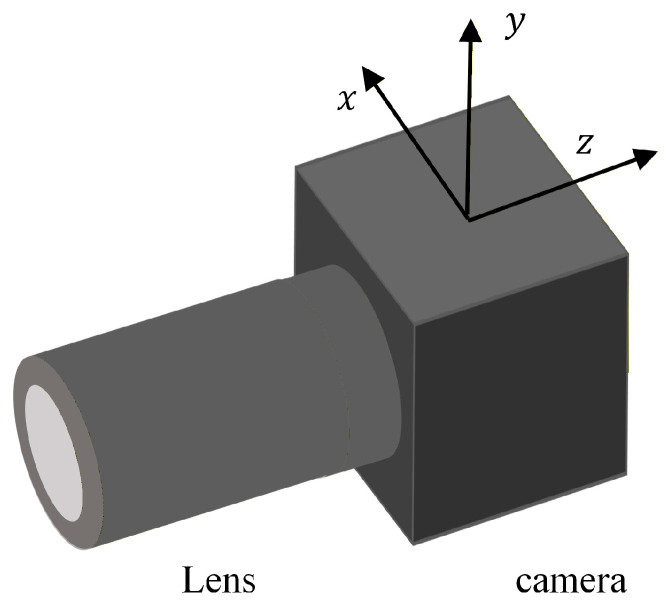
Camera motion along axis.

When employing a UAV for bridge monitoring, camera instability caused by UAV jitter can affect the accuracy of displacement monitoring results. Therefore, it is essential to compensate for camera motion in the UAV vision-based measurement system. The unstable motion of the camera on the UAV platform is divided into rotational and translational movements in the x, y, and z directions, as illustrated in Figure 3. This camera motion results in changes in the coordinates of image points on the image plane, leading to camera motion errors. These errors are classified based on different motion directions; these are *x*-axis rotation, *x*-axis translation, *y*-axis rotation, *y*-axis translation, *z*-axis rotation, and *z*-axis translation, which are respectively denoted $x_{r},x_{t},y_{r},y_{t},z_{r}$, and $z_{t}$. Among these, the camera motions that cause changes in the x-coordinate of image points on the image plane include translation along the *x*-axis, rotation around the *y*-axis, rotation around the *z*-axis, and translation along the *z*-axis. In contrast, the camera motions that induce changes in the y-coordinate of image points are rotation around the *x*-axis, translation along the *y*-axis, rotation around the *z*-axis, and translation along the *z*-axis.

**Figure 4:**
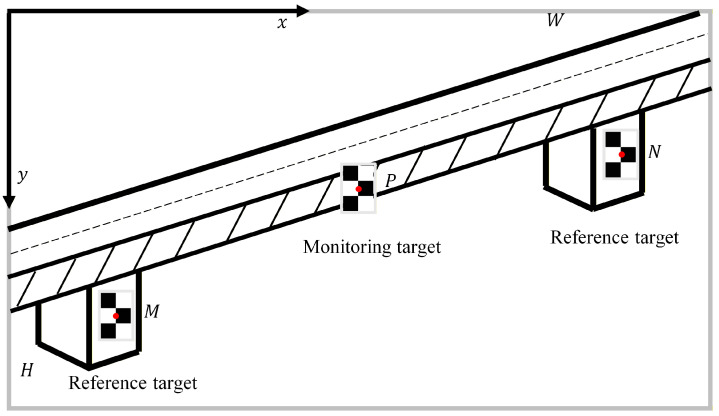
Measurement model.

During the real-time monitoring process, a measurement model is established that clearly distinguishes between two reference targets and one monitoring target on the image plane. Each target features a black-and-white chessboard pattern consisting of 3 rows and 2 columns, with two corner points for each, as depicted in Figure 4. The midpoints of the corner points for the two reference targets are labeled N and M, while their corresponding points on the image plane are represented as n and m, respectively. The midpoint of the corner points for the monitoring target is designated P, with its corresponding image point labeled p. The dimensions of the image plane are W × H, with the coordinates of its center point identified as (W/2, H/2). The displacements of points N, M, and P are computed using Equations (3)–(5):

(3) $$\left\{\begin{array}{c} D_{y}^{m}=SF_{y}^{m}(y_{t}^{m}+x_{r}^{m}+z_{r}^{m-y}+z_{t}^{m-y})\\ D_{x}^{m}=SF_{x}^{m}(y_{r}^{m}+x_{t}^{m}+z_{r}^{m-x}+z_{t}^{m-x}) \end{array}\right.$$

(4) $$\left\{\begin{array}{c} D_{y}^{n}=SF_{y}^{n}(y_{t}^{n}+x_{r}^{n}+z_{r}^{n-y}+z_{t}^{n-y})\\ D_{x}^{n}=SF_{x}^{n}(y_{r}^{n}+x_{t}^{n}+z_{r}^{n-x}+z_{t}^{n-x}) \end{array}\right.$$

(5) $$\left\{\begin{array}{c} D_{y}^{p-uc}=D_{y}^{p-c}+SF_{y}^{p}(y_{t}^{p}+x_{r}^{p}+z_{r}^{p-y}+z_{t}^{p-y})\\ D_{x}^{p-uc}=D_{x}^{p-c}+SF_{x}^{p}(y_{r}^{p}+x_{t}^{p}+z_{r}^{p-x}+z_{t}^{p-x}) \end{array}\right.$$

where $D_{y}^{p-uc}$ and $D_{x}^{p-uc}$ are the actual displacements of point P without motion error correction, and $D_{y}^{p-c}$ and $D_{x}^{p-c}$ are the true displacements of point P. $D_{y}^{n},D_{x}^{n},D_{y}^{m},$ and $D_{x}^{m}$ represent the camera motion errors of reference points N and M, respectively, along the x- and y-axes. $SF_{x}^{n},SF_{y}^{n},SF_{x}^{m},SF_{y}^{m},SF_{x}^{p},$ and $SF_{y}^{p}$ denote the horizontal x direction and vertical y direction scale factors of points *N*, *M*, and *P*, respectively. Similarly, $x_{t}^{n},x_{t}^{m},x_{t}^{p},y_{t}^{n},y_{t}^{m},$ and $y_{t}^{p}$ indicate the x-axis and y-axis translational motion errors of points N, M, and P respectively. Likewise, $x_{r}^{n},x_{r}^{m},x_{r}^{p},y_{r}^{n},y_{r}^{m},$ and $y_{r}^{p}$ signify the x-axis and y-axis rotational motion errors of points *N*, *M*, and *P* respectively. $z_{r}^{n-x},z_{r}^{n-y},z_{r}^{m-x},z_{r}^{m-y},z_{r}^{p-x},$ and $z_{r}^{p-y}$ represent the horizontal x direction and vertical y direction error components of the *z*-axis rotational motion errors of points *N*, *M*, and *P*, respectively. $z_{t}^{n-x},z_{t}^{n-y},z_{t}^{m-x},z_{t}^{m-y},z_{t}^{p-x},$ and $z_{t}^{p-y}$ are the horizontal x direction and vertical y direction error components of the *z*-axis translational motion errors of points *N*, *M*, and *P*, respectively.

#### 2.2.1. Camera Rotational Motion Around the *x*-Axis and *y*-Axis

**Figure 5:**
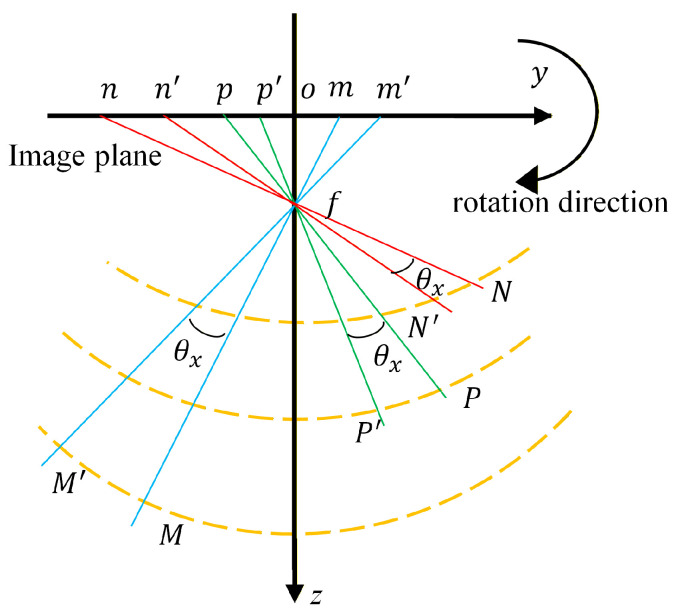
Variations in measurement points when the camera rotates around the *x*-axis.

Since the image plane is parallel to the lens plane, the law of the camera’s rotational motion around the *x*-axis is consistent with its motion around the *y*-axis. Taking the camera’s rotation around the *x*-axis as an example, this rotation introduces an error in the vertical y direction, denoted $x_{r}$. As shown in Figure 5, the *x*-axis is perpendicular to the plane of the paper and points inward. The points obtained after *N*, *M*, and *P* are rotated around the *x*-axis by an angle $\theta_{x}$. They are $N^{\prime }$, $M^{\prime }$, and $P^{\prime }$, respectively. Here, ‘o’ represents the center of the image, and ‘f’ denotes the focal length. The image points corresponding to the spatial points before and after the rotation are represented as $n\left(x_{n},y_{n}\right)$, $m\left(x_{m},y_{m}\right)$, $p\left(x_{p},y_{p}\right)$, $n^{\prime }({x^{\prime }}_{n},{y^{\prime }}_{n})$, $m^{\prime }({x^{\prime }}_{m},{y^{\prime }}_{m})$, and $p^{\prime }({x^{\prime }}_{p},{y^{\prime }}_{p})$, respectively.

Since the rotation angle $\theta_{x}$ is a small angle, the rotational motion errors of points *N*, *M*, and *P* around the *x*-axis can be calculated using Equation (6) based on geometric relationships:

(6) $$\left\{\begin{array}{c} x_{r}^{n}=|n^{\prime }f|tan\theta_{x}\\ x_{r}^{m}=|m^{\prime }f|\tan\theta_{x}\\ x_{r}^{p}=|p^{\prime }f|tan\theta_{x} \end{array}\right.$$

where $|n^{\prime }f|=f/\cos∠ofn^{\prime }$, $|m^{\prime }f|=f/\cos∠ofm^{\prime }$, and $|p^{\prime }f|=f/\cos∠ofp^{\prime }$. Among these $∠ofn^{\prime }$, $∠ofn^{\prime }$, and $∠ofn^{\prime }$ can be calculated using Equation (7), as follows:

(7) $$\left\{\begin{array}{c} ∠ofn^{\prime }=\mathrm{atan}((|y_{n}-H/2|)/f)\\ ∠ofm^{\prime }=\mathrm{atan}((|y_{m}-H/2|)/f)\\ ∠ofp^{\prime }=\mathrm{atan}((|y_{p}-H/2|)/f) \end{array}\right.$$

where f=|of|, with the unit of pixels. By comparing the rotational motion errors of points *N*, *M*, and *P*, the following expression can be obtained:

(8) $$\left\{\begin{array}{c} \frac{x_{r}^{n}}{x_{r}^{m}}=\frac{\cos\left(∠ofm^{\prime }\right)}{\cos\left(∠ofn^{\prime }\right)}\\ \frac{x_{r}^{p}}{x_{r}^{n}}=\frac{\cos\left(∠ofp^{\prime }\right)}{\cos\left(∠ofn^{\prime }\right)} \end{array}\right.$$

Similarly, for the camera’s rotational motion around the *y*-axis, the following equation is derived:

(9) $$\left\{\begin{array}{c} \frac{y_{r}^{n}}{y_{r}^{m}}=\frac{\cos\left(∠ofm^{\prime }\right)}{\cos\left(∠ofn^{\prime }\right)}\\ \frac{y_{r}^{p}}{y_{r}^{n}}=\frac{\cos\left(∠ofp^{\prime }\right)}{\cos\left(∠ofn^{\prime }\right)} \end{array}\right.$$

#### 2.2.2. Camera Rotation Around the *z*-Axis

**Figure 6:**
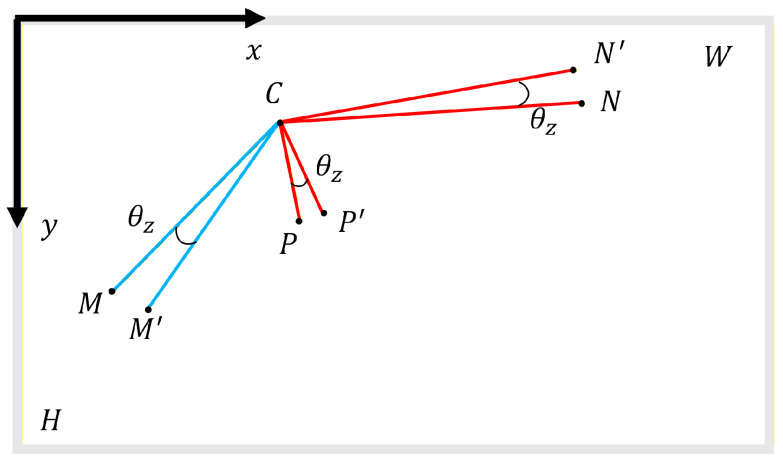
Change in measurement points upon rotating the camera around the *z*-axis.

Rotation of the camera around the *z*-axis introduces displacement errors in both the x and y directions. The image point errors resulting from this type of rotation occur only within the image plane, allowing for analysis within this plane. As shown in Figure 6, $C\left(x_{c},y_{c}\right)$ in the image plane is the center of rotation. After rotating around the *z*-axis by an angle $\theta_{z}$, the spatial points *N*, *M*, and *P* become $N^{\prime }$, $M^{\prime }$, and $P^{\prime }$, respectively, along with their corresponding image points before and after rotation, which are indicated as $n\left(x_{n},y_{n}\right)$, $m\left(x_{m},y_{m}\right)$, $p\left(x_{p},y_{p}\right)$, $n^{\prime }({x^{\prime }}_{n},{y^{\prime }}_{n})$, $m^{\prime }({x^{\prime }}_{m},{y^{\prime }}_{m})$, and $p^{\prime }({x^{\prime }}_{p},{y^{\prime }}_{p})$.

When rotating counterclockwise by an angle $\theta_{z}$, the following equation can be derived based on geometric relationships:

(10) $$\left\{\begin{array}{c} \left[\begin{array}{c} {x^{\prime }}_{n}\\ {y^{\prime }}_{n} \end{array}\right]=\left[\begin{array}{cc} \cos\theta_{z} & \sin\theta_{z}\\ -\sin\theta_{z} & \cos\theta_{z} \end{array}\right]\left[\begin{array}{c} x_{n}-x_{c}\\ y_{n}-y_{c} \end{array}\right]+\left[\begin{array}{c} x_{c}\\ y_{c} \end{array}\right]\\ \left[\begin{array}{c} {x^{\prime }}_{m}\\ {y^{\prime }}_{m} \end{array}\right]=\left[\begin{array}{cc} \cos\theta_{z} & \sin\theta_{z}\\ -\sin\theta_{z} & \cos\theta_{z} \end{array}\right]\left[\begin{array}{c} x_{m}-x_{c}\\ y_{m}-y_{c} \end{array}\right]+\left[\begin{array}{c} x_{c}\\ y_{c} \end{array}\right]\\ \left[\begin{array}{c} {x^{\prime }}_{p}\\ {y^{\prime }}_{p} \end{array}\right]=\left[\begin{array}{cc} \cos\theta_{z} & \sin\theta_{z}\\ -\sin\theta_{z} & \cos\theta_{z} \end{array}\right]\left[\begin{array}{c} x_{p}-x_{c}\\ y_{p}-y_{c} \end{array}\right]+\left[\begin{array}{c} x_{c}\\ y_{c} \end{array}\right] \end{array}\right.$$

The image point displacements of points *N*, *M*, and *P* can be mathematically represented as follows:

(11) $$\left\{\begin{array}{c} \left[\begin{array}{c} z_{r}^{n-x}\\ z_{r}^{n-y} \end{array}\right]=\left[\begin{array}{c} {x^{\prime }}_{n}-x_{n}\\ {y^{\prime }}_{n}-y_{n} \end{array}\right]=\left[\begin{array}{c} \left(\cos\theta_{z}-1)(x_{n}-x_{c})+\sin\theta_{z}(y_{n}-y_{c}\right)\\ \left(\cos\theta_{z}-1)(y_{n}-y_{c})-\sin\theta_{z}(x_{n}-x_{c}\right) \end{array}\right]\\ \left[\begin{array}{c} z_{r}^{m-x}\\ z_{r}^{m-y} \end{array}\right]=\left[\begin{array}{c} {x^{\prime }}_{m}-x_{m}\\ {y^{\prime }}_{m}-y_{m} \end{array}\right]=\left[\begin{array}{c} \left(\cos\theta_{z}-1)(x_{m}-x_{c})+\sin\theta_{z}(y_{m}-y_{c}\right)\\ \left(\cos\theta_{z}-1)(y_{m}-y_{c})-\sin\theta_{z}(x_{m}-x_{c}\right) \end{array}\right]\\ \left[\begin{array}{c} z_{r}^{p-x}\\ z_{r}^{p-y} \end{array}\right]=\left[\begin{array}{c} {x^{\prime }}_{p}-x_{p}\\ {y^{\prime }}_{p}-y_{p} \end{array}\right]=\left[\begin{array}{c} \left(\cos\theta_{z}-1)(x_{p}-x_{c})+\sin\theta_{z}(y_{p}-y_{c}\right)\\ \left(\cos\theta_{z}-1)(y_{p}-y_{c})-\sin\theta_{z}(x_{p}-x_{c}\right) \end{array}\right] \end{array}\right.$$

Subtracting the first term from the second term in Equation (11) yields the following expression:

(12) $$\left[\begin{array}{c} z_{r}^{m-x}-z_{r}^{n-x}\\ z_{r}^{m-y}-z_{r}^{n-y} \end{array}\right]=\left[\begin{array}{c} \left(\cos\theta_{z}-1)(x_{m}-x_{n})+\sin\theta_{z}(y_{m}-y_{n}\right)\\ \left(\cos\theta_{z}-1)(y_{m}-y_{n})-\sin\theta_{z}(x_{m}-x_{n}\right) \end{array}\right]$$

Similarly, subtracting the first term from the third term in Equation (12) leads to

(13) $$\left[\begin{array}{c} z_{r}^{p-x}-z_{r}^{n-x}\\ z_{r}^{p-y}-z_{r}^{n-y} \end{array}\right]=\left[\begin{array}{c} \left(\cos\theta_{z}-1)(x_{p}-x_{n})+\sin\theta_{z}(y_{p}-y_{n}\right)\\ \left(\cos\theta_{z}-1)(y_{p}-y_{n})-\sin\theta_{z}(x_{p}-x_{n}\right) \end{array}\right]$$

Likewise, when rotating clockwise by an angle $\theta_{z}$, Equations (14) and (15) are obtained based on geometric relationships:

(14) $$\left[\begin{array}{c} z_{r}^{m-x}-z_{r}^{n-x}\\ z_{r}^{m-y}-z_{r}^{n-y} \end{array}\right]=\left[\begin{array}{c} \left(\cos\theta_{z}-1)(x_{m}-x_{n})-\sin\theta_{z}(y_{m}-y_{n}\right)\\ \left(\cos\theta_{z}-1)(y_{m}-y_{n})+\sin\theta_{z}(x_{m}-x_{n}\right) \end{array}\right]$$

(15) $$\left[\begin{array}{c} z_{r}^{p-x}-z_{r}^{n-x}\\ z_{r}^{p-y}-z_{r}^{n-y} \end{array}\right]=\left[\begin{array}{c} \left(\cos\theta_{z}-1)(x_{p}-x_{n})-\sin\theta_{z}(y_{p}-y_{n}\right)\\ \left(\cos\theta_{z}-1)(y_{p}-y_{n})+\sin\theta_{z}(x_{p}-x_{n}\right) \end{array}\right]$$

According to Equations (12)–(15), the differences in image point displacements between measurement points caused by *z*-axis rotation are independent of the center of rotation.

#### 2.2.3. Camera Translational Motion Along the *x*-Axis and *y*-Axis

**Figure 7:**
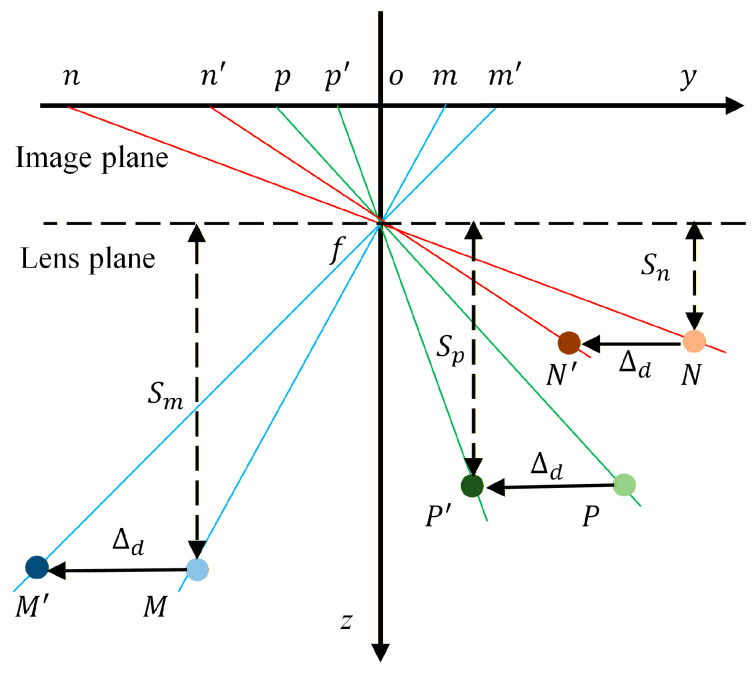
Measurement point change of camera translation along the y-axis.

The translational motion of the camera is analyzed along the *y*-axis. As illustrated in Figure 7, the translation of the camera along this axis only affects the y-coordinates of the measurement points, with the translation magnitude along the *y*-axis being $\Delta_{d}$. After translation, the spatial points *N*, *M*, and *P* are transformed into $N^{\prime }$, $M^{\prime }$, and $\;P^{\prime }$, respectively. Here, o represents the center of the image, *f* is the focal length, and f refers to the principal optical axis of the lens. The corresponding image points for the spatial points before and after translation are indicated as $n\left(x_{n},y_{n}\right)$, $m\left(x_{m},y_{m}\right)$, $p\left(x_{p},y_{p}\right)$, $n^{\prime }({x^{\prime }}_{n},{y^{\prime }}_{n})$, $m^{\prime }({x^{\prime }}_{m},{y^{\prime }}_{m})$, and $p^{\prime }({x^{\prime }}_{p},{y^{\prime }}_{p})$, respectively. The translational motion errors along the *y*-axis for points N, M, and P can be calculated using Equation (16):

(16) $$\left\{\begin{array}{c} y_{t}^{n}=|on^{\prime }|-|on|\\ y_{t}^{m}=|om^{\prime }|-|om|\\ y_{t}^{p}=|op^{\prime }|-|op| \end{array}\right.$$

From the geometric relationship, the ratios of the *y*-axis translational motion errors between reference point *N* and reference point *M* and between reference point *N* and monitoring point *P* can be obtained as follows:

(17) $$\left\{\begin{array}{c} \frac{y_{t}^{n}}{y_{t}^{m}}=\frac{\Delta_{d}S_{m}/f}{\Delta_{d}S_{n}/f}=\frac{SF_{y}^{m}}{SF_{y}^{n}}\\ \frac{y_{t}^{n}}{y_{t}^{p}}=\frac{\Delta_{d}S_{p}/f}{\Delta_{d}S_{n}/f}=\frac{SF_{y}^{p}}{SF_{y}^{n}} \end{array}\right.$$

where $S_{n}$, $S_{m}$, and $S_{p}$ are the actual distances from points *N*, *M*, and *P* to the lens plane, respectively; $SF_{y}^{N}$, $SF_{y}^{M}$, and $SF_{y}^{P}$ are the vertical y direction scale factors of points *N*, *M*, and *P*, respectively. Similarly, the following expression can be used to determine the camera’s translation along the *x*-axis:

(18) $$\left\{\begin{array}{c} \frac{x_{t}^{n}}{x_{t}^{m}}=\frac{SF_{x}^{m}}{SF_{x}^{n}}\\ \frac{x_{t}^{n}}{x_{t}^{p}}=\frac{SF_{x}^{p}}{SF_{x}^{n}} \end{array}\right.$$

#### 2.2.4. Camera Translation Along the *z*-Axis

**Figure 8:**
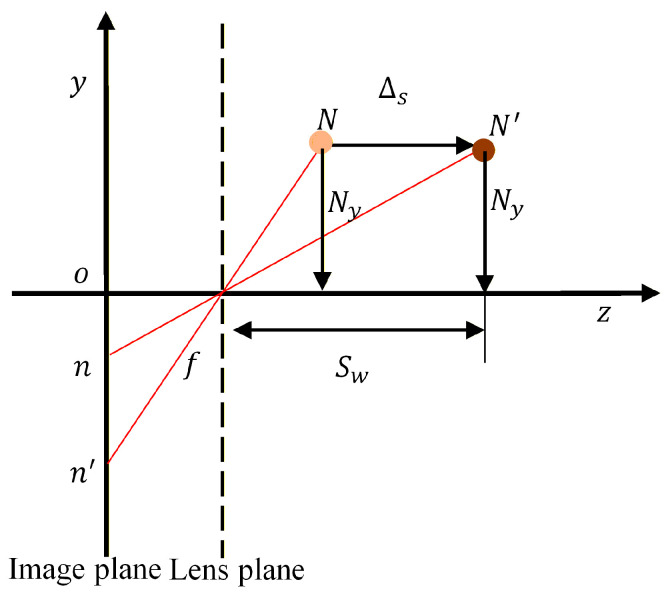
Variation in the y-coordinate of measurement point N when the camera is translated along the *z*-axis.

Translation of the camera along the *z*-axis alters the distance from the monitoring point to the lens plane, which manifests as displacement errors in both the x and y directions on the image plane. To understand this effect, a change in the y-coordinate of point *N* due to the camera’s translation is considered along the *z*-axis. As shown in Figure 8, assume the camera moves along the *z*-axis by a magnitude of $\Delta_{s}$, and the distance from point N to the *z*-axis is defined as $N_{y}$. After translation, the spatial point N shifts accordingly to $N^{\prime }$. Here, $S_{w}$ denotes the distance from $N^{\prime }$ to the optical center of the lens. The corresponding image points of spatial point N before and after translation are represented by $n\left(x_{n},y_{n}\right)$ and $n^{\prime }({x^{\prime }}_{n},{y^{\prime }}_{n})$, respectively.

Based on the geometric relationship, the following equation can be derived:

(19) $$z_{t}^{n-y}=|on^{\prime }|-|on|$$

where $z_{t}^{n-y}$ is the displacement error of point N in the y direction, which is caused by *z*-axis translation. To resolve Equation (19), the unit pixel displacement variation is defined as $\Delta_{zy}^{n-y}$, which can be determined using the following expression:

(20) $$\Delta_{zt}^{n-y}=\frac{\left|on^{\prime }|-|on\right|}{\left|on\right|}$$

Rearranging this equation yields Equation (21):

(21) $$\Delta_{zt}^{n-y}=\frac{|on^{\prime }|/N_{y}-|on|/N_{y}}{|on|/N_{y}}=\frac{1/SF_{y}^{n^{\prime }}-1/SF_{y}^{n}}{1/SF_{y}^{n}}=\frac{SF_{y}^{n}-SF_{y}^{n^{\prime }}}{SF_{y}^{n^{\prime }}}$$

The displacement error associated with a spatial point N is then computed using the following expression:

(22) $$z_{t}^{n-y}=\Delta_{zt}^{n-y}|on|$$

Consequently, the error components for the *z*-axis translational motion along the vertical y direction at points *N*, *M*, and *P* can be determined as follows:

(23) $$\left\{\begin{array}{c} z_{t}^{n-y}=\Delta_{zy}^{n-y}|on{|}_{y}\\ \begin{array}{c} z_{t}^{m-y}=\Delta_{zy}^{m-y}|om{|}_{y}\\ z_{t}^{p-y}=\Delta_{zy}^{p-y}|op{|}_{y} \end{array} \end{array}\right.$$

Similarly, the mathematical expression for error components of the *z*-axis translational movement along the horizontal x direction for points N, M, and P is given as follows:

(24) $$\left\{\begin{array}{c} z_{t}^{n-x}=\Delta_{zx}^{n-x}|on{|}_{x}\\ \begin{array}{c} z_{t}^{m-x}=\Delta_{zx}^{m-x}|om{|}_{x}\\ z_{t}^{p-x}=\Delta_{zx}^{p-x}|op{|}_{x} \end{array} \end{array}\right.$$

where $|on{|}_{y},|om{|}_{y}$, and $|om{|}_{y}$ represent the differences between the y-coordinates of image points, while $|on{|}_{x},|om{|}_{x},|om{|}_{x}$ represent the differences between the x-coordinates of image points n, m, p, and W/2. By defining the scale change as $SF_{y}^{n}-SF_{y}^{n^{\prime }}$, it can be concluded from Equation (21) that the scale change is independent of the measurement points, meaning the scale factors for each measurement point affected by translational motion along the *z*-axis change synchronously.

Based on the error patterns, this paper designs a stepwise error correction scheme as follows: initially, the rotational error along the *z*-axis is corrected, followed by rectification of errors in *z*-axis translation. Thereafter, the differences observed during *x*-axis rotation and *y*-axis translation are resolved systematically. The specific method steps are detailed in the upcoming sections.

(1) Correction of *z*-axis Rotation Error

In the measurement model, for the vertically aligned reference target N, the x-coordinates of its two corner points $n_{1}$ and $n_{2}$ are nearly equal. Consequently, the error terms $y_{r}^{n_{1}}$ and $y_{r}^{n_{2}}$ in the x direction displacements of these two points are also equal. Additionally, since the two corner points on the same target share the identical scale factor, the error terms $z_{t}^{n_{1}-x}$ and $z_{t}^{n_{2}-x}$ as well as $x_{t}^{n_{1}}$ and $x_{t}^{n_{2}}$ in the x direction displacements of these two points are equal as well. Therefore, by calculating the difference between the x-displacements of the two points, only the error term caused by *z*-axis rotation is retained in the determined value. Given that the *z*-axis rotation angle $\theta_{z}$ is small, and $\left(\cos\theta_{z}-1\right)$ <<$\sin\theta_{z}$, Equation (13) can be simplified as follows:

(25) $$\sin\theta_{z}=(z_{r}^{n_{2}-x}-z_{r}^{n_{1}-x})/(y_{n_{2}}-y_{n_{1}})$$

The *z*-axis rotation angle $\theta_{z}$ can be solved using Equation (25). Further, Equations (26) and (27) for the error components $z_{r}^{m-x},z_{r}^{p-x},z_{r}^{m-y},z_{r}^{p-y}$ of the *z*-axis rotational motion errors of points M and P are derived. Similarly, the derivation process for the clockwise rotation angle $\theta_{z}$ is the same and will not be repeated here:

(26) $$\left[\begin{array}{c} z_{r}^{m-x}\\ z_{r}^{m-y} \end{array}\right]=\left[\begin{array}{c} \left(\cos\theta_{z}-1)(x_{m}-x_{n})-\sin\theta_{z}(y_{m}-y_{n}\right)\\ \left(\cos\theta_{z}-1)(y_{m}-y_{n})+\sin\theta_{z}(x_{m}-x_{n}\right) \end{array}\right]+\left[\begin{array}{c} z_{r}^{n-x}\\ z_{r}^{n-y} \end{array}\right]$$

(27) $$\left[\begin{array}{c} z_{r}^{p-x}\\ z_{r}^{p-y} \end{array}\right]=\left[\begin{array}{c} \left(\cos\theta_{z}-1)(x_{p}-x_{n})-\sin\theta_{z}(y_{p}-y_{n}\right)\\ \left(\cos\theta_{z}-1)(y_{p}-y_{n})+\sin\theta_{z}(x_{p}-x_{n}\right) \end{array}\right]+\left[\begin{array}{c} z_{r}^{n-x}\\ z_{r}^{n-y} \end{array}\right]$$

(2) Correction of *z*-axis Translation Error

To rectify the *z*-axis translational motion error, it is necessary to calculate the variation in unit pixel displacement using Equation (21). Since the scale change is consistent across various measurement points, the correction accuracy is enhanced by calculating the scale change using the nearest reference point. Subsequently, a variation in the unit pixel displacement is computed. For instance, if the scale change is calculated using target *N*, then the unit pixel displacement variations $\Delta_{zt}^{p-x}$ and $\Delta_{zt}^{p-y}$ for the monitoring point *P* will be calculated. Finally, Equations (23) and (24) are used to compute the *z*-axis translational motion error terms $z_{t}^{p-y}$ and $z_{t}^{p-x}$ for the monitoring point *P*.

(3) Correction of x- and y-axis Rotation and Translation errors

After correcting the *z*-axis rotation and translation errors, only rotational and translational motion errors in the x- and y-axes remain for the monitoring point P. At this point, Equations (3)–(5) can be simplified to Equations (28)–(30):

(28) $$\left\{\begin{array}{c} {\hat{D}}_{y}^{n}=SF_{y}^{n}(y_{t}^{n}+x_{r}^{n})\\ {\hat{D}}_{x}^{n}=SF_{x}^{n}(y_{r}^{n}+x_{t}^{n}) \end{array}\right.$$

(29) $$\left\{\begin{array}{c} {\hat{D}}_{y}^{m}=SF_{y}^{m}(y_{t}^{m}+x_{r}^{m})\\ {\hat{D}}_{x}^{m}=SF_{y}^{m}(y_{r}^{m}+x_{t}^{m}) \end{array}\right.$$

(30) $$\left\{\begin{array}{c} {\hat{D}}_{y}^{p-uc}=D_{y}^{p-c}+SF_{y}^{p}(y_{t}^{p}+x_{r}^{p})\\ {\hat{D}}_{x}^{p-uc}=D_{x}^{p-c}+SF_{x}^{p}(y_{r}^{p}+x_{t}^{p}) \end{array}\right.$$

In these equations, ${\hat{D}}_{y}^{p-uc}$ and ${\hat{D}}_{x}^{p-uc}$ are the actual displacements of point *N* and *M* after correcting for the *z*-axis rotation and translation errors, which are known values. Similarly, $\;D_{y}^{p-c}$ and $\;D_{x}^{p-c}$ represent the actual displacement of point P, which is an unknown value. The actual displacements of reference points *N* and *M* after correcting for the *z*-axis rotation error are denoted ${\hat{D}}_{y}^{n},{\hat{D}}_{x}^{n},{\hat{D}}_{y}^{m},{\hat{D}}_{x}^{m}$, and these are also known values. In fact, all scale factors are established as known values in this work. The solution process is outlined as follows.

First, Equations (8), (17), (28), and (29) are combined to solve for $x_{r}^{n}$, $y_{t}^{n}$, $x_{r}^{m}$, $y_{t}^{m}$, $x_{r}^{p}$ and $y_{t}^{p}$.

Second, Equations (9), (18), (28), and (29) are integrated to derive $y_{r}^{n}$, $x_{t}^{n}$, $y_{r}^{m}$, $x_{t}^{m}$,$y_{r}^{p}$ and $x_{t}^{p}$.

Thereafter, $x_{r}^{p}$, $x_{t}^{p}$, $y_{r}^{p}$ and $y_{t}^{p}$ are substituted into Equation (30) to determine $D_{y}^{p-c}$ and $D_{x}^{p-c}$. At this point, the displacement result of the monitoring point is obtained after correcting the 3D motion errors.

## 3. Experiment

### 3.1. Method Validation

#### 3.1.1. Experimental Protocol

The UAV-based vision method is effective for scenarios where deploying fixed monitoring equipment is challenging. To assess the reliability of the proposed method, displacement monitoring experiments were conducted using the UAV, focusing on the side of a building as the monitoring target. The instruments and equipment utilized in the experiment are detailed in Table 2.

**Figure 9:**
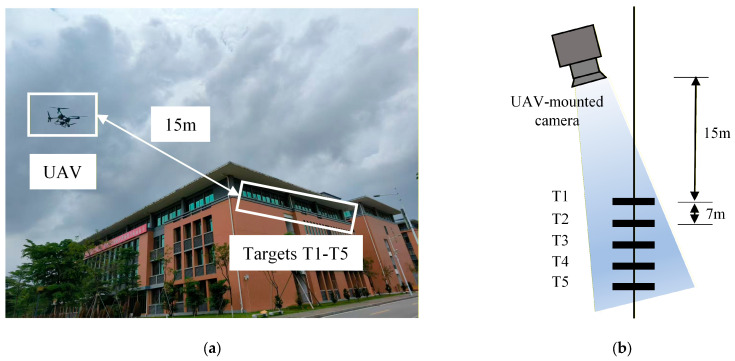

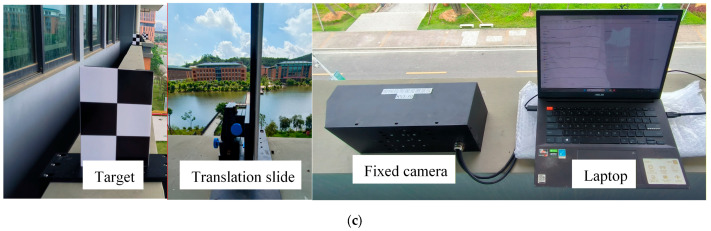
Monitoring scene and field deployment: (**a**) monitoring scene; (**b**) experimental setup; (**c**) target, translation slide, and fixed camera field deployment.

**Figure 10:**
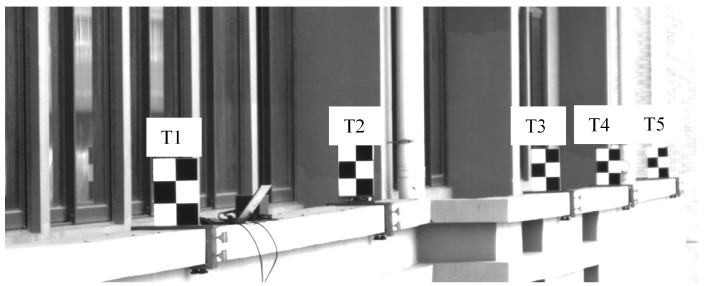
UAV-mounted camera imaging results.

During the experiment, five targets were placed at equal intervals of 7 m along the outer side of the windowsill on the fourth floor of the building, numbered T1 to T5 from left to right. A translational sliding table was installed at the base of target T2 to simulate actual displacement. To obtain a true reference value for the displacement of target T2, a fixed camera was positioned 2 m away for synchronous monitoring. Figure 9a depicts the UAV monitoring scenario, while Figure 9b illustrates the experimental setup. Figure 9c shows the on-site arrangement of the targets, translation slide, and fixed camera. Figure 10 displays the on-site images captured by the UAV-mounted camera.

During the experiment, the acquisition frequency of both the UAV-mounted camera and the fixed camera was set to 100 frames per second. It lasts for 20 s. Target T2 was moved vertically by translating the slide table. Following the method described in Section 2.2, targets T1 and T5 were used as reference targets in this experiment, while targets T2, T3, and T4 were used as monitoring targets. Due to significant vibrations in the UAV system, some targets were lost in the camera’s field of view, resulting in a total of 1695 valid images being collected.

#### 3.1.2. Experimental Results and Analysis

**Figure 11:**
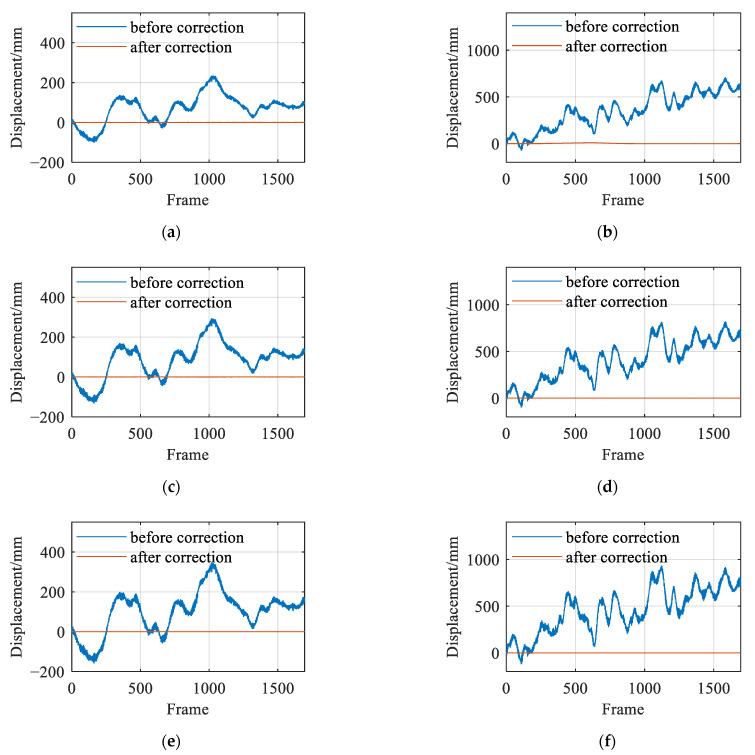
Displacement curve before and after motion error correction of UAV-mounted camera: (**a**) horizontal x displacement curve of target T2; (**b**) vertical y displacement curve of target T2; (**c**) horizontal x displacement curve of target T3; (**d**) vertical y displacement curve of target T3; (**e**) horizontal x displacement curve of target T4; (**f**) vertical y displacement curve of target T4.

Using the method proposed in this study, the experimental data were processed, and the results are presented in Figure 11. The findings show that the displacement results for targets T2, T3, and T4 approached zero after incorporating the proposed correction schemes. This suggests that the proposed method effectively rectified the errors encountered during the camera movement on UAV platforms in outdoor conditions.

**Figure 12:**
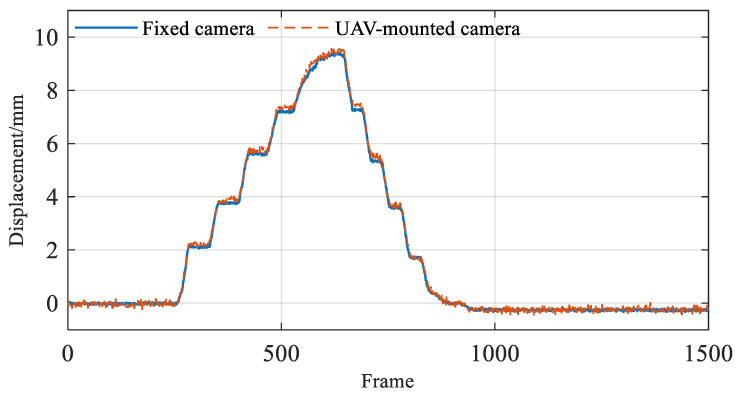
Comparison of monitoring results of the UAV-mounted camera and fixed camera.

Furthermore, the corrected T2 target displacement monitoring results were compared with those measured by the fixed camera, matching a total of 1500 images, as shown in Figure 12. The comparison results indicate a high consistency between the monitoring results from the UAV-mounted camera and the fixed camera, which intuitively reinforces the measurement accuracy of the proposed method. To quantitatively evaluate measurement errors, root mean square error (RMSE) statistical analysis was conducted on the results obtained, with specific statistical details presented in Table 3.

### 3.2. Monitoring of Lunzhou Bridge

#### 3.2.1. Overview

**Figure 13:**
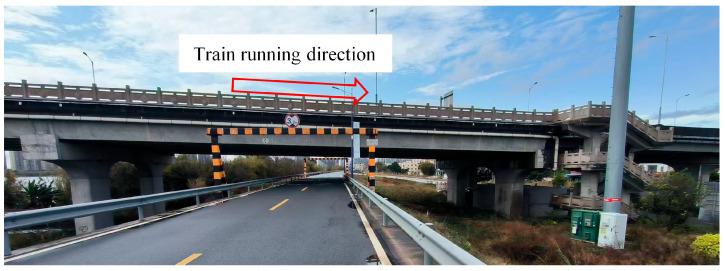
Lunzhou Highway Bridge in China.

To further assess the performance of the UAV-based vision system for monitoring displacement, experiments were conducted on the Lunzhou Bridge, as illustrated in Figure 13. The Lunzhou Bridge is situated in Qingyuan City, Guangdong Province, China. It serves as a vital cross-river passage that connects both sides of the Beijiang River, extending from Qingfei Highway in the northwest to Beijiang East Road in the southeast. Monitoring the displacement of the Lunzhou Bridge is essential for verifying the adaptability and accuracy of the UAV platform system in engineering applications. Additionally, it provides valuable data to support the structural safety assessment and long-term operation and maintenance of highway bridges. Furthermore, to evaluate the accuracy of the UAV-based monitoring system, a multi-point displacement measurement method employing a Scheimpflug camera, as proposed by Xing [24], was used for comparison.

#### 3.2.2. Monitoring Points

**Figure 14:**
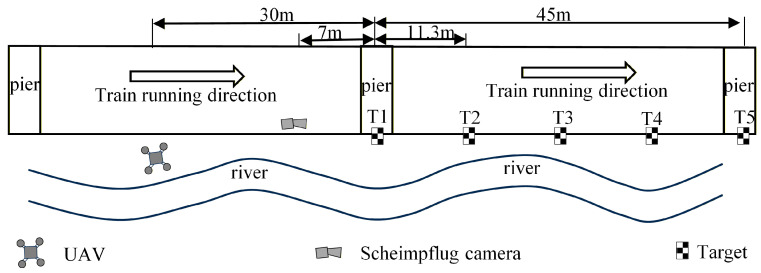
Monitoring points of Lunzhou Highway Bridge.

In this article, a motion correction method is explored for UAVs that is similar to the multi-point displacement measurement approach using a Scheimpflug camera (MindVision, Shenzhen, China) [22]. Both methods require the use of two reference targets. Following these considerations, specific road sections were monitored, as sketched in Figure 14. The bridge section being investigated is 45 m long, equipped with five targets strategically placed along the pedestrian guardrail. Targets T1 and T5 are positioned above the bridge pier and serve as reference points. In contrast, targets T2, T3, and T4 are located 11.3 m, 22.5 m, and 33.8 m away from the bridge pier, respectively. The Scheimpflug camera is installed at a horizontal distance of 7 m from the nearest bridge pier, enabling comparison of the measurement results with those obtained from the UAV vision-based displacement monitoring system. This UAV system is deployed on the side of the bridge, hovering at a horizontal distance of 30 m from the nearest pier.

#### 3.2.3. Monitoring Implementation

**Figure 15:**
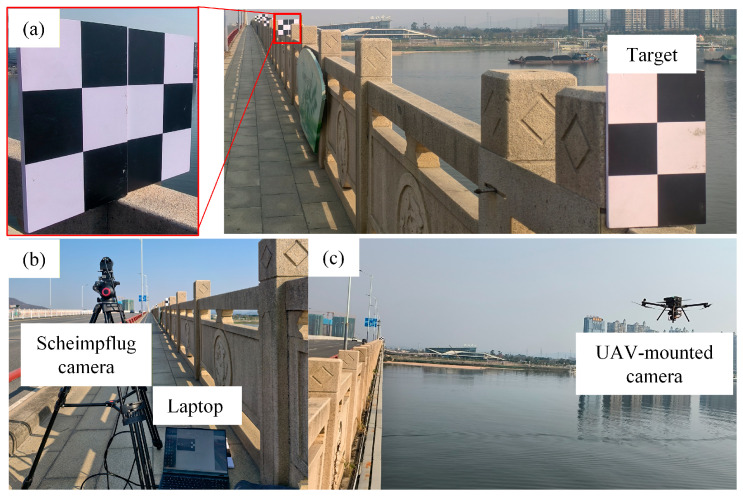
Installation location and site layout. (**a**) Targets (**b**) Scheimpflug camera arrangement (**c**). UAV deployment.

Initially, the targets were arranged according to the configuration shown in Figure 14. The corresponding images of the targets at different locations on the bridge are shown in Figure 15a. To avoid mutual occlusion within the camera’s field of view, two targets were installed at each of the points T2 through T5. The target size L observed by the UAV-mounted camera at points T1 to T5 was 100, 100, 100, 150, and 150 mm, while the target size L recorded by the fixed platform camera at T1 to T5 was 100, 100, 100, 100, and 120 mm.

**Figure 16:**
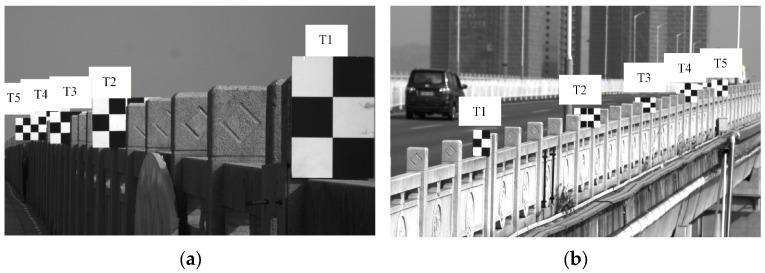
Images of target locations obtained using the (**a**) Scheimpflug camera (**b**) UAV-mounted camera.

Upon positioning the targets, the bridge camera and UAV vision measurement system were deployed, as shown in Figure 15b,c, respectively. Figure 16a illustrates the imaging results from the Scheimpflug camera, while Figure 16b displays the imaging results obtained from the UAV-mounted camera. Thereafter, data collection and processing were executed with a frame rate set to 100 frames per second for both the fixed platform and the UAV-mounted camera, with a total collection time of 15 sec. When processing data from the UAV platforms, the accuracy of corner positioning was hindered by the long measurement distance, leading to significant noise in the scale factor calculation and substantial noise levels in the *z*-axis translation motion error results. To mitigate this, a median filtering method with a filtering radius of 100 was applied to denoise the scale factor calculations. Since the bridge predominantly undergoes vertical deformation during vehicle traffic, the subsequent analysis focuses on the vertical displacement monitoring results.

#### 3.2.4. Monitoring Results and Analysis

**Figure 17:**
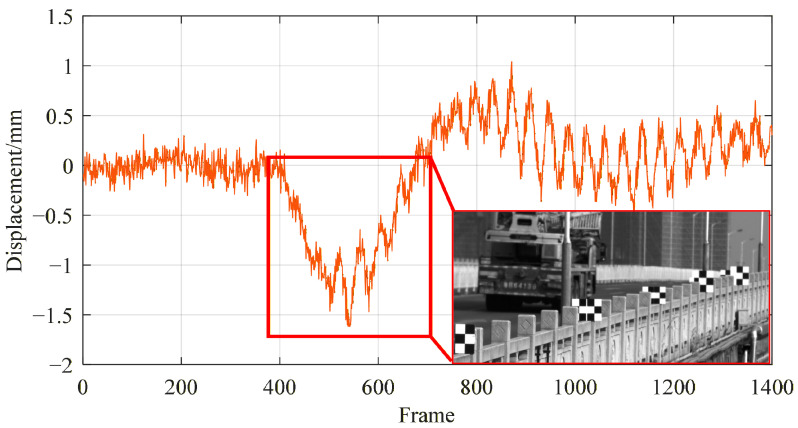
Displacement curve of midspan target T3.

According to the method described in Section 2.2, in this experiment, targets T1 and T5 were used as reference targets, while targets T2, T3, and T4 were used as monitoring targets. Due to significant vibrations of the drone system, some targets were lost in the camera’s field of view, and a total of 1400 valid images were collected. The obtained image data was compared and analyzed with the displacement results of the mid span target T3 in this article’s system. These results are shown in Figure 17.

**Figure 18:**
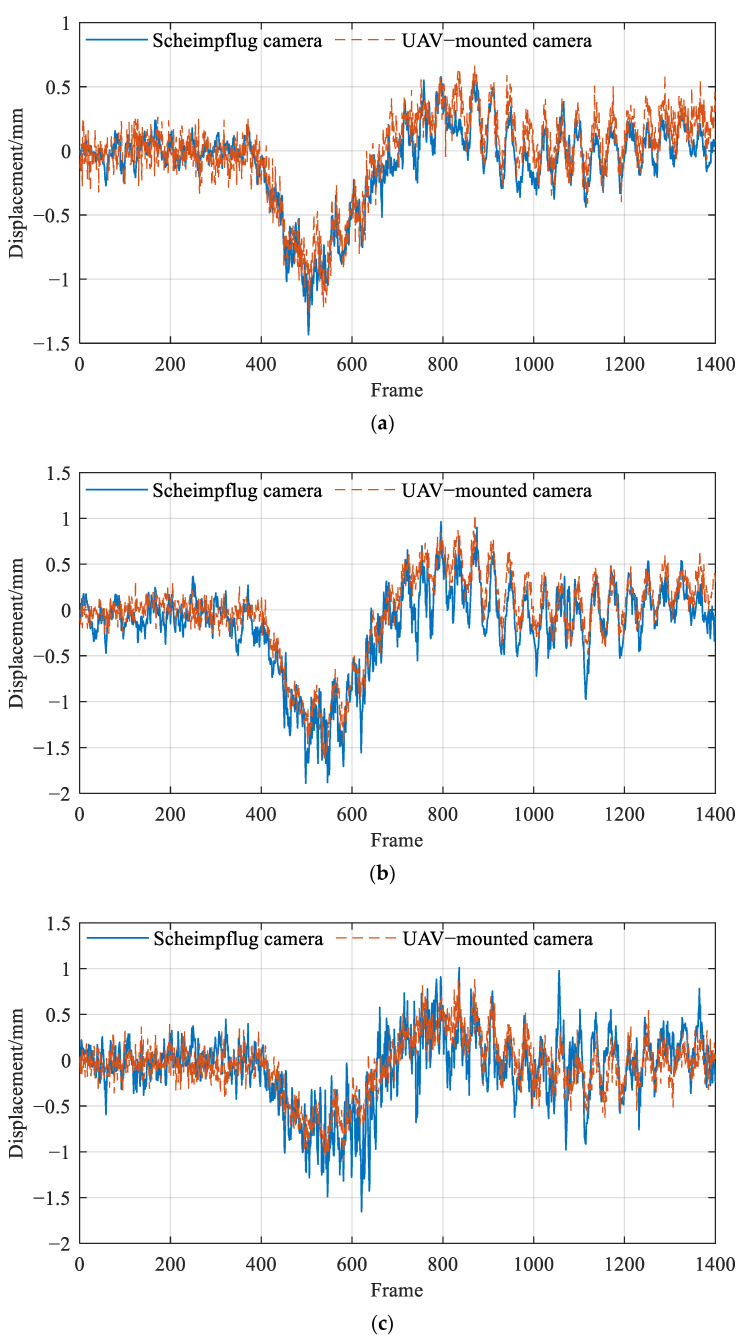
Comparison of measurement results between the UAV-mounted camera and Scheimpflug camera for (**a**) target T2, (**b**) target T3, and (**c**) target T4.

As evident from Figure 18, when the vehicle crosses the bridge, it exhibits a noticeable downward deflection trend, which aligns with theoretical expectations. This finding initially confirms the reliability of the UAV vision-based displacement measurement system. Furthermore, a comparison was made between the displacement monitoring results from the fixed-platform camera and the UAV-mounted camera. The results are displayed in Figure 18, which indicates that the displacement monitoring outcomes from both platforms are highly consistent, thereby validating the accuracy of the UAV vision-based system in monitoring the displacement of the bridge.

Based on the comparative analysis presented in Figure 18, it can be observed that the vertical displacement at target T3, located at the mid-span of the bridge, was the largest, exceeding 1.5 mm. The vertical displacements at targets T2 and T4 were both approximately 1 mm. It is worth noting that the displacement at T4 was slightly smaller, which may be attributed to the longer monitoring distance, resulting in reduced image resolution and measurement accuracy. In addition, the monitoring results for targets T3 and T4 obtained from the fixed-platform camera exhibited significant fluctuations. This behavior can mainly be ascribed to two factors: first, potential stray light interference caused by the shading cloth enclosing the connection between the Scheimpflug camera and the lens, and second, the relatively small aperture of the lens, which limited the amount of incoming light and led to a lower signal-to-noise ratio (SNR) in the acquired data.

To quantitatively evaluate the accuracy of displacement measurements obtained from the UAV-mounted camera, the results from a fixed-platform camera were used as reference values. The RMSE of the UAV-based vision system was calculated. The specific findings are tabulated in Table 4. It was observed that, at a measurement distance of 75 m, the RMSE for vertical displacement monitoring results improved to less than 0.3 mm. This indicates that the UAV-mounted camera can maintain a high level of measurement accuracy even during long-distance monitoring, confirming the precision of the UAV vision-based displacement monitoring system.

**Figure 19:**
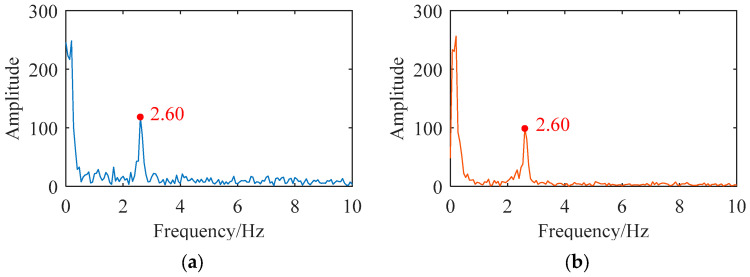
Spectrum analysis and comparison of measurement results of the (**a**) Scheimpflug camera and (**b**) UAV-mounted camera.

Furthermore, a spectral analysis was performed on the measurement results obtained from both the Scheimpflug camera and the UAV-mounted camera, as illustrated in Figure 19. The results revealed that both cameras yielded a similar dominant frequency of 2.60 Hz, which further validates the high accuracy of the displacement measurement system obtained using the UAV-mounted camera.

## 4. Discussion

The proposed UAV vision-based multi-point displacement measurement method and system resolve the low-accuracy issue of traditional UAV vision monitoring caused by UAV jitter. Verified against a Scheimpflug camera, field tests on the Lunzhou Highway Bridge achieved full-span vertical multi-point displacement monitoring with an RMSE < 0.3 mm. The results were highly consistent with those of the Scheimpflug camera, meeting the sub-millimeter to millimeter-level precision requirements of bridge dynamic displacement monitoring.

Spectral analysis showed that both the UAV system and the Scheimpflug camera identified the bridge’s vertical dominant response frequency as 2.60 Hz, confirming the UAV system’s capability to capture structural dynamic characteristics for bridge safety assessments (e.g., structural integrity evaluation via frequency variations). Compared with the fixed Scheimpflug camera (7 m from the nearest pier), the UAV can hover 30 m away and flexibly adjust position to avoid obstacles, showing superior adaptability to complex on-site environments.

The proposed method has universal technical principles and can be extended to other bridge types (e.g., cable-stayed bridges, steel truss bridges) and complex environments by replacing adaptive targets and adjusting algorithm parameters. Supplementing additional bridge case studies or numerical simulations will further improve the system’s generalizability and robustness, which will be the focus of future research.

Despite verified accuracy, this study has limitations. First, the 15 s (100 fps) data collection was designed to capture the bridge’s high-frequency dynamic response rather than long-term static drift. Although the UAV has a 25 min full-load endurance, long-term monitoring for algorithm verification will be conducted in future research. Notably, long-term monitoring requires upgraded UAV hardware (e.g., enhanced power supply and communication for on-board computing) and improved algorithm stability during prolonged operation, both of which represent feasible future directions.

Second, the small-angle approximation in Section 2.2 is a limitation, as wind-induced UAV attitude fluctuations may exceed its effective range. Future improvements include equipping a three-axis gimbal (stabilizing roll, pitch, yaw) and optimizing the algorithm to reduce reliance on this approximation. Third, to enhance vibration robustness, a visual positioning module will be integrated to assist the RTK system in real-time attitude adjustment, improving target tracking stability under variable conditions. Finally, the method’s geometric assumptions need in-depth analysis; while planar motion of the UAV and target minimizes the impact of camera tilt angle (via scale factor), UAV z-direction movement exacerbates perpendicularity assumption errors. Although scale factor compensation was applied, the sensitivity of accuracy to the impacts of tilt angle and comprehensive geometric deviation requires systematic exploration. Future research will focus on analyzing these impacts and optimizing coupling error compensation strategies for tilt angle and z-direction movement.

In summary, the proposed UAV vision system achieves high consistency with the Scheimpflug camera and accurately captures the bridge’s 2.60 Hz dominant frequency. Despite limitations (short monitoring duration, small-angle approximation constraints, and insufficient geometric assumption analysis), its flexible deployment offers significant advantages, making it practical for bridge displacement monitoring. Future work will focus on extending monitoring duration, optimizing attitude stability, enhancing vibration robustness, and exploring geometric assumption impacts with optimized coupling error compensation to improve practical value.

## 5. Conclusions

This paper proposes a UAV vision-based method for multi-point bridge displacement measurement and a corresponding monitoring system (integrating UAV, industrial camera, computing terminal, and measurement targets). Field tests on the Lunzhou Highway Bridge (Qingyuan, Guangdong) yielded key findings, detailed as follows. (1) The system successfully captures multi-point dynamic displacement data, with results highly consistent with those of a Scheimpflug camera (maximum RMSE < 0.3 mm). (2) The proposed method effectively mitigates UAV jitter-induced errors, improving monitoring accuracy. (3) The vertical dominant response frequency of the Lunzhou Highway Bridge is 2.60 Hz.

For broader implications in bridge health monitoring, the system enriches non-contact monitoring technologies by virtue of its flexible deployment. It overcomes the limitations of fixed devices (e.g., Scheimpflug cameras) in complex environments, providing reliable multi-point dynamic displacement data for safety assessment. Additionally, multi-target synchronous measurement reduces single-point monitoring costs, enhancing the economic feasibility of large-scale applications.

In summary, the proposed UAV vision system achieves high consistency with the Scheimpflug camera (RMSE < 0.3 mm) and accurately captures the bridge’s 2.60 Hz dominant frequency. Despite limitations (short monitoring duration, small-angle approximation constraints, and insufficient geometric assumption analysis), its flexible deployment provides significant advantages for practical bridge displacement monitoring. Future work will focus on extending monitoring duration, optimizing attitude stability, enhancing vibration robustness, and exploring the impacts of geometric assumption with optimized coupling error compensation to improve practical value.

## Figures and Tables

**Table 1:** UAV vision-based measurement system parameters.

Equipment	Specifications
UAV-mounted camera	Pixel size: 5.86 μm Resolution: 1920 × 1200 Acquisition frequency: ≤165 FPS Focal length: 85 mm
Computing terminal	CPU: Core i3-N305 RAM: 32 G
Targets	Size: 3 L × 2 L
UAV	Maximum payload: 5 kg Full-load endurance: 25 min Positioning accuracy: ≤0.1 m

**Table 2:** UAV vision-based measurement experimental equipment.

Equipment	Parameter	Quantity
UAV-mounted camera	Resolution: 1920 × 1200 Focal length: 85 mm	1
Fixed camera	Resolution: 720 × 540 Focal length: 8 mm	1
Steel ruler	3 m	1
Target and scaffold	Size: 20 × 30 cm	5
Translation slide table	Travel range: 0–3 cm	1

**Table 3:** RMSE statistics of displacement measurement results of the UAV-mounted camera monitoring experiment (mm).

Target Number	Horizontal x-Direction Displacement			Vertical y-Direction Displacement		
	Before Correction	After Correction	RMSE Improvement Rate (%)	Before Correction	After Correction	RMSE Improvement Rate (%)
T2	99.82	0.14	99	365.77	0.11	99
T3	123.57	0.21	99	462.58	0.19	99
T4	144.91	0.15	99	516.08	0.25	99

**Table 4:** RMSE statistics of vertical displacement measurement results of Lunzhou Highway Bridge (mm).

Target Number	Vertical Displacement Measurement Result
T2	0.18
T3	0.25
T4	0.29

## Data Availability

Data are available on request due to restrictions (e.g., privacy, legal, or ethical reasons).
